# The Interplay Between Retirement Transition Sequences and Mental Health: Focusing on Gender Differences

**DOI:** 10.1093/geroni/igab046.3145

**Published:** 2021-12-17

**Authors:** Oejin Shin, Sojung Park, BoRin Kim

**Affiliations:** 1 University of Illinois at Urbana Champaign, Savoy, Illinois, United States; 2 Washington university, St.Louis, Missouri, United States; 3 University of New Hampshire, University of New Hampshire, New Hampshire, United States

## Abstract

Although retirement has been given a substantial amount of attention, there are gaps in the literature on 1) the various forms of retirement (nature= voluntary/involuntary, timing= early/ late, type= full/ partial) using with previous employment history, and 2) gender differences in retirement transition. Drawing on the life course perspective, this study examined the gender differences in retirement transition sequences using the labor participation history and various forms of retirement. Data are from the 2004 to 2016 HRS with 1,653 older workers. Sequence analysis was used to answer how individuals experienced retirement in the extended time frame. OLS regression analysis was used to estimate the relationship between retirement transition sequences and depression. For both genders, eight clusters of retirement transition sequences were identified. However, the most prevalent group for males was those who experience voluntary retirement transition from full-time work in mid-time point (19%), while the most prevalent group was a gradual involuntary retirement (21%) for females. Regarding the association with depression, those who experienced voluntary retirement after full-time work in all different time points (early, mid, late) and those who retired from self-employment were less likely to have depressive symptoms for males. For females, only those who experienced voluntary retirement from full-time work in mid-time point were less likely to have depressive symptoms. This result contributes to identifying the heterogeneity of retirement transition sequences and their association with depression. The result suggests important implications of gender-specified intervention programs to prevent involuntary retirement and mental health support program for involuntary retirees.

